# A multi-channel CRISPR-based method for rapid, sensitive detection of four diseases of *Brassica rapa* in the field

**DOI:** 10.1093/hr/uhae351

**Published:** 2024-12-12

**Authors:** Xiaojing Liu, Tongbing Su, Xiaoyun Xin, Peirong Li, Weihong Wang, Cancan Song, Xiuyun Zhao, Deshuang Zhang, Yangjun Yu, Jiao Wang, Ning Li, Miao Wang, Fenglan Zhang, Shuancang Yu

**Affiliations:** State Key Laboratory of Vegetable Biobreeding, Beijing Vegetable Research Center (BVRC), Beijing Academy of Agriculture and Forestry Science (BAAFS), No. 50, Zhanghua Road, Haidian District, Beijing 100097, China; Key Laboratory of Biology and Genetic Improvement of Horticultural Crops (North China), Ministry of Agriculture, No. 50, Zhanghua Road, Haidian District, Beijing 100097, China; Beijing Key Laboratory of Vegetable Germplasm Improvement, No. 50, Zhanghua Road, Haidian District, Beijing 100097, China; National Engineering Research Center for Vegetables, No. 50, Zhanghua Road, Haidian District, Beijing 100097, China; State Key Laboratory of Vegetable Biobreeding, Beijing Vegetable Research Center (BVRC), Beijing Academy of Agriculture and Forestry Science (BAAFS), No. 50, Zhanghua Road, Haidian District, Beijing 100097, China; Key Laboratory of Biology and Genetic Improvement of Horticultural Crops (North China), Ministry of Agriculture, No. 50, Zhanghua Road, Haidian District, Beijing 100097, China; Beijing Key Laboratory of Vegetable Germplasm Improvement, No. 50, Zhanghua Road, Haidian District, Beijing 100097, China; National Engineering Research Center for Vegetables, No. 50, Zhanghua Road, Haidian District, Beijing 100097, China; State Key Laboratory of Vegetable Biobreeding, Beijing Vegetable Research Center (BVRC), Beijing Academy of Agriculture and Forestry Science (BAAFS), No. 50, Zhanghua Road, Haidian District, Beijing 100097, China; Key Laboratory of Biology and Genetic Improvement of Horticultural Crops (North China), Ministry of Agriculture, No. 50, Zhanghua Road, Haidian District, Beijing 100097, China; Beijing Key Laboratory of Vegetable Germplasm Improvement, No. 50, Zhanghua Road, Haidian District, Beijing 100097, China; National Engineering Research Center for Vegetables, No. 50, Zhanghua Road, Haidian District, Beijing 100097, China; State Key Laboratory of Vegetable Biobreeding, Beijing Vegetable Research Center (BVRC), Beijing Academy of Agriculture and Forestry Science (BAAFS), No. 50, Zhanghua Road, Haidian District, Beijing 100097, China; Key Laboratory of Biology and Genetic Improvement of Horticultural Crops (North China), Ministry of Agriculture, No. 50, Zhanghua Road, Haidian District, Beijing 100097, China; Beijing Key Laboratory of Vegetable Germplasm Improvement, No. 50, Zhanghua Road, Haidian District, Beijing 100097, China; National Engineering Research Center for Vegetables, No. 50, Zhanghua Road, Haidian District, Beijing 100097, China; State Key Laboratory of Vegetable Biobreeding, Beijing Vegetable Research Center (BVRC), Beijing Academy of Agriculture and Forestry Science (BAAFS), No. 50, Zhanghua Road, Haidian District, Beijing 100097, China; Key Laboratory of Biology and Genetic Improvement of Horticultural Crops (North China), Ministry of Agriculture, No. 50, Zhanghua Road, Haidian District, Beijing 100097, China; Beijing Key Laboratory of Vegetable Germplasm Improvement, No. 50, Zhanghua Road, Haidian District, Beijing 100097, China; National Engineering Research Center for Vegetables, No. 50, Zhanghua Road, Haidian District, Beijing 100097, China; Beijing Yishi Biotech Co., Ltd., No. 36 Chuangye Middle Road, Haidian District, Beijing 100085, China; State Key Laboratory of Vegetable Biobreeding, Beijing Vegetable Research Center (BVRC), Beijing Academy of Agriculture and Forestry Science (BAAFS), No. 50, Zhanghua Road, Haidian District, Beijing 100097, China; Key Laboratory of Biology and Genetic Improvement of Horticultural Crops (North China), Ministry of Agriculture, No. 50, Zhanghua Road, Haidian District, Beijing 100097, China; Beijing Key Laboratory of Vegetable Germplasm Improvement, No. 50, Zhanghua Road, Haidian District, Beijing 100097, China; National Engineering Research Center for Vegetables, No. 50, Zhanghua Road, Haidian District, Beijing 100097, China; State Key Laboratory of Vegetable Biobreeding, Beijing Vegetable Research Center (BVRC), Beijing Academy of Agriculture and Forestry Science (BAAFS), No. 50, Zhanghua Road, Haidian District, Beijing 100097, China; Key Laboratory of Biology and Genetic Improvement of Horticultural Crops (North China), Ministry of Agriculture, No. 50, Zhanghua Road, Haidian District, Beijing 100097, China; Beijing Key Laboratory of Vegetable Germplasm Improvement, No. 50, Zhanghua Road, Haidian District, Beijing 100097, China; National Engineering Research Center for Vegetables, No. 50, Zhanghua Road, Haidian District, Beijing 100097, China; State Key Laboratory of Vegetable Biobreeding, Beijing Vegetable Research Center (BVRC), Beijing Academy of Agriculture and Forestry Science (BAAFS), No. 50, Zhanghua Road, Haidian District, Beijing 100097, China; Key Laboratory of Biology and Genetic Improvement of Horticultural Crops (North China), Ministry of Agriculture, No. 50, Zhanghua Road, Haidian District, Beijing 100097, China; Beijing Key Laboratory of Vegetable Germplasm Improvement, No. 50, Zhanghua Road, Haidian District, Beijing 100097, China; National Engineering Research Center for Vegetables, No. 50, Zhanghua Road, Haidian District, Beijing 100097, China; State Key Laboratory of Vegetable Biobreeding, Beijing Vegetable Research Center (BVRC), Beijing Academy of Agriculture and Forestry Science (BAAFS), No. 50, Zhanghua Road, Haidian District, Beijing 100097, China; Key Laboratory of Biology and Genetic Improvement of Horticultural Crops (North China), Ministry of Agriculture, No. 50, Zhanghua Road, Haidian District, Beijing 100097, China; Beijing Key Laboratory of Vegetable Germplasm Improvement, No. 50, Zhanghua Road, Haidian District, Beijing 100097, China; State Key Laboratory of Vegetable Biobreeding, Beijing Vegetable Research Center (BVRC), Beijing Academy of Agriculture and Forestry Science (BAAFS), No. 50, Zhanghua Road, Haidian District, Beijing 100097, China; Key Laboratory of Biology and Genetic Improvement of Horticultural Crops (North China), Ministry of Agriculture, No. 50, Zhanghua Road, Haidian District, Beijing 100097, China; Beijing Key Laboratory of Vegetable Germplasm Improvement, No. 50, Zhanghua Road, Haidian District, Beijing 100097, China; National Engineering Research Center for Vegetables, No. 50, Zhanghua Road, Haidian District, Beijing 100097, China; Institute of Quality Standardization & Testing Technology for Agro-Products, Chinese Academy of Agricultural Sciences, No. 12, Zhongguancun South Street, Haidian District, Beijing 100081, China; Key Laboratory of Agrofood Safety and Quality (Beijing), Ministry of Agriculture and Rural Areas, No.12, Zhongguancun South Street, Haidian District, Beijing 100081, China; State Key Laboratory of Vegetable Biobreeding, Beijing Vegetable Research Center (BVRC), Beijing Academy of Agriculture and Forestry Science (BAAFS), No. 50, Zhanghua Road, Haidian District, Beijing 100097, China; Key Laboratory of Biology and Genetic Improvement of Horticultural Crops (North China), Ministry of Agriculture, No. 50, Zhanghua Road, Haidian District, Beijing 100097, China; Beijing Key Laboratory of Vegetable Germplasm Improvement, No. 50, Zhanghua Road, Haidian District, Beijing 100097, China; National Engineering Research Center for Vegetables, No. 50, Zhanghua Road, Haidian District, Beijing 100097, China; State Key Laboratory of Vegetable Biobreeding, Beijing Vegetable Research Center (BVRC), Beijing Academy of Agriculture and Forestry Science (BAAFS), No. 50, Zhanghua Road, Haidian District, Beijing 100097, China; Key Laboratory of Biology and Genetic Improvement of Horticultural Crops (North China), Ministry of Agriculture, No. 50, Zhanghua Road, Haidian District, Beijing 100097, China; Beijing Key Laboratory of Vegetable Germplasm Improvement, No. 50, Zhanghua Road, Haidian District, Beijing 100097, China; National Engineering Research Center for Vegetables, No. 50, Zhanghua Road, Haidian District, Beijing 100097, China

## Abstract

Pathogens significantly restrict the production of *Brassica rapa* (*B. rapa* L. ssp. Pekinensis), with climate change and evolving planting patterns exacerbating disease prevalence. Multichannel rapid diagnostic methods in the field can facilitate the early detection and control of diseases in *B. rapa*. Here, we established a multichannel lateral flow biosensor (LFB) combined with a CRISPR/Cas12a cleavage assay for the simultaneous detection of four *B. rapa* diseases. Key innovations of this study include: (1) High specificity and sensitivity, down to pathogen concentrations of 1.5 pg/μl—due to the optimization of crRNA secondary structure: the more stable the crRNA, the higher its detection sensitivity. (2) Optimized visual detection parameters. We identified ideal concentration ratios for the visual fluorescence detection system: 50 nM Cas12a, 50 nM crRNA, and 500 nM ssDNA fluorescent probe. Furthermore, the optimal concentrations of components on the LFB detection system were 3 μl SA-GNPs, 500 nM ssDNA test strip probe, 0.5 mg/ml biotin-BSA as the test line, and 1 mg/ml anti-FITC as the control line. (3) Field-Ready Cas-AIRPA Platform. We developed the on-site Cas-AIRPA platform for the simultaneous detection of *B. rapa* pathogens by combining rapid nucleic acid extraction and a four-channel lateral flow biosensor (4-LFB), which quickly provides disease-related information through a specific 2D barcode. Analysis of *B. rapa* samples in the field confirmed the suitability of the Cas-AIRPA platform for rapid (~25 min) and simultaneous on-site detection of four diseases of *B. rapa*. This platform can also be adapted to detect other plant diseases in the field.

## Introduction

*Brassica rapa* includes a variety of economically important oil and vegetable crops, such as oilseeds, leafy vegetables, and turnips. *Brassica rapa* is one of the most significant vegetable crops in China. However, its production is often hindered by four prevalent diseases: downy mildew, verticillium wilt, black rot, and club root [1–4]. Downy mildew, caused by *Peronospora parasitica* (Pers.) Fr., is particularly concerning due to its wide distribution, high incidence, and rapid onset, which can affect *B. rapa* throughout its growth period, severely impacting both yield and quality [5]. Black rot, a bacterial disease caused by *Xanthomonas campestris* pv. campestris (Pammel) Dowson, primarily affects vascular bundles through systemic infection [6]. Verticillium wilt is primarily induced by *Verticillium dahliae* and *Verticillium longisporum*, manifesting as yellowing leaves, stunted growth, and discoloration of vascular bundles [7]. Club root, caused by *Plasmodiophora brassicae* Woronin, a fungus, results in the proliferation of parenchyma cells in the roots, leading to tumor formation [7].

The severity of these diseases is closely related to the abundance and virulence of the pathogens involved. Rapid diagnosis in the field is crucial for the early detection of pathogens and control of *B. rapa* diseases [8]. Existing methods based on nucleic acid detection include polymerase chain reaction (PCR) [9], multiplex PCR [10, 11], droplet digital PCR (ddPCR) [12], and real-time fluorescence quantitative PCR [13–15]. These techniques offer high specificity and sensitivity in bacterial detection but are hindered by their reliance on expensive equipment, limiting their field applicability. Loop-mediated isothermal amplification (LAMP) [16–19] and recombinase polymerase amplification (RPA) [20, 21] can be performed at room temperature and are compatible with field use, but their detection sensitivity is relatively low. Similarly, the gene chip method [22, 23] has the advantage of high throughput, but the detection sensitivity is low and the technology is expensive. Protein-based detection methods strongly depend on the preparation and screening of pathogen-specific antibodies [24]. Overall, traditional detection methods are limited by their complexity, cost, and practicality, highlighting the need for more efficient and straightforward detection approaches for *B. rapa* pathogens.

Recently, new methods leveraging the trans-cleavage activity of CRISPR/Cas nucleases have been developed for the detection of various pathogens in diverse crops [25, 26]. The most widely used type II CRISPR/Cas systems for nucleic acid detection include type V Cas12 [27–29] and type VI Cas13 [30–32]. When the target nucleic acid is present, CRISPR RNA (crRNA) specifically binds to the target sequence, activating the trans-cleavage activity of Cas12 or Cas13 and resulting in the degradation of ssDNA or ssRNA probes. Various CRISPR/Cas-based nucleic acid detection methods have been developed, as exemplified by SHERLOCK [30], HOLMES [33], DETECTR [34], and other detection platforms. Several CRISPR/Cas12 nucleic acid detection methods employing gold immunochromatographic assays have been utilized to quickly detect plant diseases without the need for complex instruments [19]. However, these methods are focused on single-pathogen detection and have yet to be tested in field conditions.

**Figure 1:**
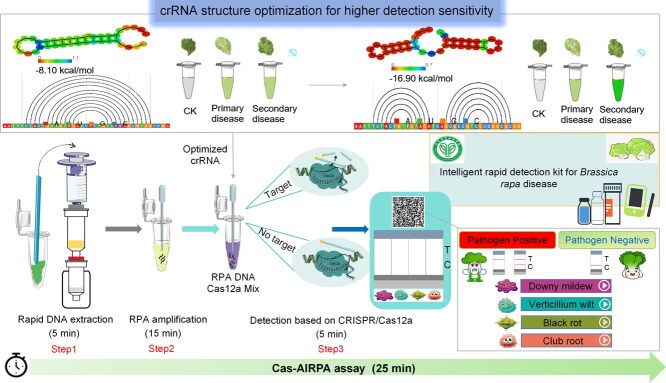
Design of the Cas-AIRPA platform. Cas-AIRPA employs three closely linked steps: DNA preparation, RPA, and detection based on CRISPR/Cas12a cleavage and the 4-LFB test. Prior to detection, the structures of the crRNAs were optimized: healthy leaves of the same age and diseased leaves at different stage for detection tests, in order to identify the crRNAs with the highest sensitivity for subsequent use. The color scale reflects the stability of the crRNA secondary structure. When the colorimetric value of the crRNA secondary structure is close to 0, it indicates low energy and greater stability, which corresponds to higher detection sensitivity. In the third step, the Cas12a/crRNA complex recognizes the PAM (Protospacer adjacent motif, PAM) site and cleaves the amplified RPA products, and the ssDNA probe is digested by the trans-cleavage activity of Cas12a. The results are read using a fluorescence reporter or 4-LFB. The disease-specific 2D barcode provides diagnostic information about the disease; with additional content accessible through the barcode, as shown in the Supplementary Video.

In this study, we optimized the crRNA structure in the CRISPR/Cas system to enhance the trans-cleavage activity of the nuclease and validated its sensitivity base on symptoms severity. Using this optimized crRNA, we developed an RPA-assisted CRISPR/FnCas12a artificial intelligence assay (Cas-AIRPA), a rapid, effective, and multiplexed detection method capable of simultaneously identifying four common *B. rapa* diseases—downy mildew, verticillium wilt, black rot, and club root—in both laboratory and field settings. The Cas-AIRPA platform integrates rapid nucleic acid extraction with a portable four-channel lateral flow biosensor (4-LFB) and a disease-specific 2D barcode that can be scanned via a mobile phone for quick field detection. Designed to be highly sensitive, user-friendly, and cost-effective, this platform enables the simultaneous detection of multiple pathogens, retained in the prediction and management of *B. rapa* diseases.

## Results

### Schematic illustration of the Cas-AIRPA platform

The Cas-AIRPA platform (Fig. 1) utilizes the principle of RPA isothermal amplification technology and the characteristics of CRISPR/Cas12a with *in vitro* trans-cleavage activity. The detection platform consists of three steps. The first step involves the rapid extraction of nucleic acids from *B. rapa*, as described by Wang Lab [35]. The *B. rapa* leaves are placed into a 1.5-ml Eppendorf (EP) tube, where they are crushed with a stirring stick, and the sample is lysed using lysis buffer. The lysis mixture is added to the device for DNA extraction; the entire extraction process takes only 5 min to obtain DNA of high concentration and purity (Fig. S1) and does not require any equipment. The second step is to perform an RPA reaction using the extracted DNA; the amplified product is generated within 15 min at 37°C. The third step is to visually detect the amplified product using 4-LFB. The Cas12a/crRNA/dsDNA ternary complex activates the trans-cleavage activity of Cas12a, resulting in the cleavage of the ssDNA fluorescent probe and subsequent generation of a fluorescent signal. The fluorescent signal generated from trans-cleavage is monitored by a fluorescence detector or the 4-LFB. For the 4-LFB, we also developed a disease-specific 2D barcode that can be scanned by a mobile phone to access essential information, disease symptoms, pathogens, photographs of disease symptoms, crRNA sequences for the detection site, and the rDNA-ITS sequences of the pathogens (Fig. 1). Prior to detection, we optimized the crRNA structure using the RNA fold web server to obtain higher detection sensitivity.

In summary, we developed the Cas-AIRPA kit for the simultaneous detection of four diseases in the field based on the trans-cleavage activity of CRISPR/Cas12a, RPA technology, 4-LFB, and the use of a mobile phone. The kit contains: 1. A rapid DNA extraction device and reagents (EP tube, adsorption column, filter column, dropper, and three bottles of extraction reagent); 2. RPA reagents (EP tube, two bottles of amplification reagent); 3. Premixed buffer for Cas12a detection (crRNAs for the four diseases, one bottle of detection mixture); and 4. A 4-LFB for detection. Using this kit, DNA can be extracted from leaves in the field to detect four diseases of *B. rapa* within 25 min.

### Improving the sensitivity of the Cas-AIRPA assay

**Figure 2:**
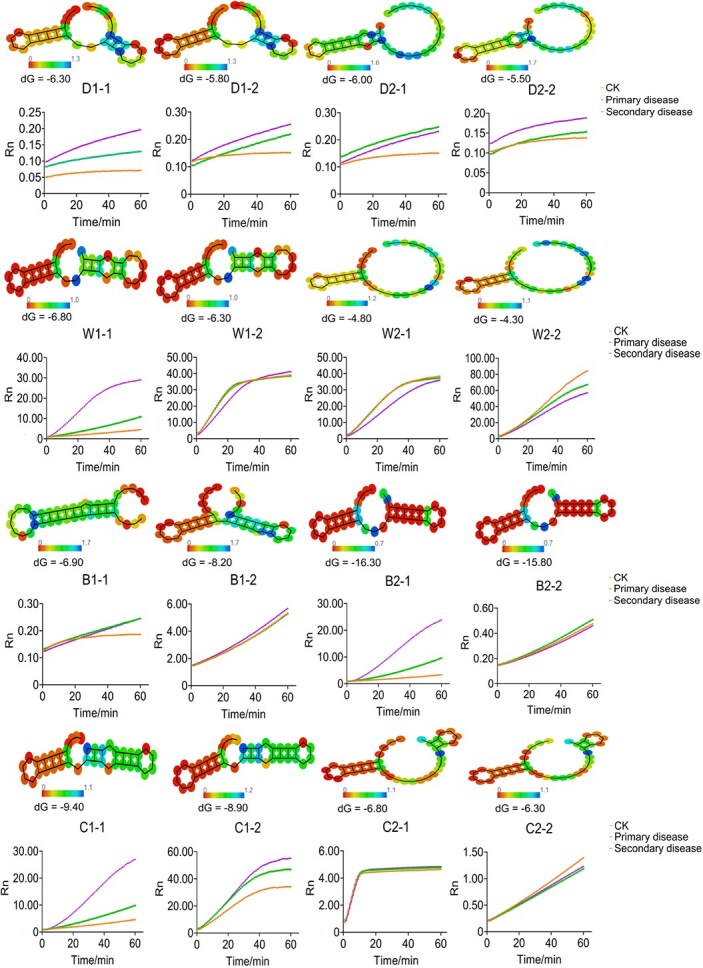
Optimization of crRNA structure to detect four *B. rapa* diseases. Four crRNA structures were designed to detect each disease (downy mildew: D1–1, D1–2, D2–1, D2–2; verticillium wilt: W1–1, W1–2, W2–1, W2–2; black rot: B1–1, B1–2, B2–1, B2–2; club root: C1–1, C1–2, C2–1, C2–2), and the optimal crRNA structure was selected by predicting the structural stability of the crRNAs using the RNA fold web server and performing fluorescent signal detection experiments. When the colorimetric value of the secondary structure of crRNA is close to 0, the crRNA secondary structure is more stable. dG energy prediction was performed using RNA Folding Form V2.3. The smaller the dG energy value, the more stable the secondary structure of the crRNA. In the fluorescence detection experiment, leaves with different levels of disease severity (primary disease and secondary disease) and leaves without disease (CK) were selected for detection. The fluorescent signals were collected every minute for 60 min. Rn is the ratio of the reported fluorescence value of the dye (FAM) to the fluorescence value of the inert reference dye (ROX II). All data were produced from three technical replicates, and data are shown as mean ± SD.

To improve the sensitivity of the Cas-AIRPA assay, we optimized the structures of the crRNAs. As shown in Fig. 2, we predicted the stability of the secondary structures of 16 crRNAs synthesized *in vitro* using the RNA fold web server and calculated the dG energy values using RNA Folding Form V2.3. We also conducted fluorescence visualization experiments on the 16 crRNA structures and simultaneously selected leaves with no disease symptoms (CK) and different disease grades (primary and secondary) to verify the sensitivity of the Cas-AIRPA system. Among the crRNAs used to detect downy mildew, verticillium wilt, black rot, and club root, D1–1 (downy mildew), W1–1 (verticillium wilt), B2–1 (black rot), and C1–1 (club root) had the lowest energy values (dG = −6.30, dG = −6.80, dG = −16.30, and dG = −9.40, respectively), and their colorimetric values were closest to red, indicating that these structures were the most stable. The secondary structures of D1–2, D2–1, and D2–2 for downy mildew; W1–2, W2–1, and W2–2 for verticillium wilt; B1–1, B1–2, and B2–2 for black rot, and C1–2, C2–1, and C2–2 for club root had relatively high energy values, and their colorimetric values tended toward green, indicating that these structures are unstable. Fluorescence detection experiments also showed that D1–1, W1–1, B2–1, and C1–1 could successfully distinguish different levels of disease severity (no disease, primary disease, and secondary disease). With increasing reaction time, the increase in the fluorescent signal for plants with secondary disease was greater than that for those with primary disease, and the increase in the fluorescent signal for plants with primary disease was greater than that for plants with no disease symptoms (CK). We ultimately selected D1–1, W1–1, B2–1, and C1–1 for the detection of the four diseases of *B. rapa*.

### Optimal conditions for the fluorescence visual assay

**Figure 3:**
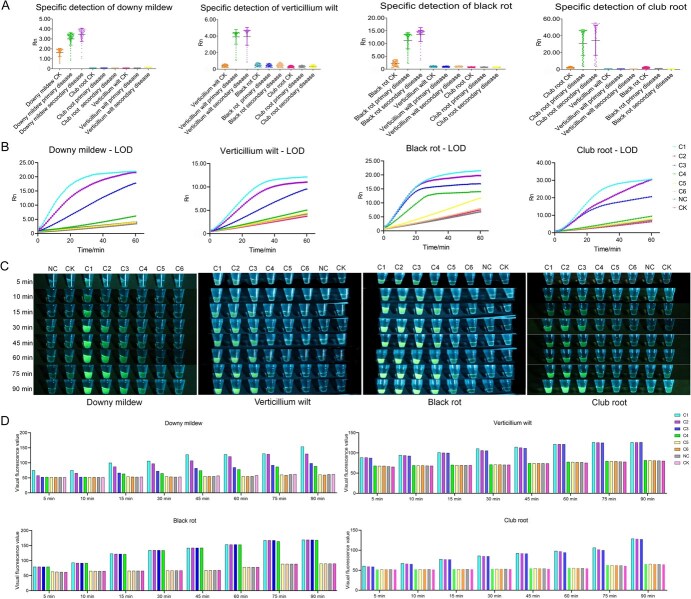
Fluorescence visualization platform for detecting four *B. rapa* diseases. (A) Specificity of the four-disease assay. In each test, the crRNA of the target pathogen was used to detect the target pathogen and two other pathogens. The fluorescent signal was monitored and collected every minute for a total of 60 min. (B) Sensitivity of the four-disease assay. In sensitivity tests, each pathogen was prepared at concentrations of 15 (C1), 1.5 (C2), 0.15 (C3), 0.015 (C4), 0.0015 (C5), and 0.00015 ng/μl (C6); NC is the negative control, with H_2_O used in place of the DNA template. The DNA template used in CK was extracted from *B. rapa* leaves without disease. (C) Visual results of the four-disease assay. Fluorescent signals were monitored, and photographs were taken at 5, 10, 15, 30, 45, 60, 75, and 90 min. (D) Digitized results of the four-disease assay. All data were produced from three technical replicates, and data are shown as mean ± SD.

After optimizing the crRNAs, we validated the detection activity and specificity of the CRISPR/Cas12a system. As shown in Fig. S2, when only two out of three components (Cas12a, crRNA, and target dsDNA) were included to the system, the fluorescent signal was significantly lower than when all three components were present, indicating that the formation of the Cas12a/crRNA/target dsDNA ternary complex activates the trans-cleavage activity of Cas12a. As shown in Fig. 3a, when the DNAs of both target and nontarget pathogens were introduced to the detection system for downy mildew, verticillium wilt, black rot, or club root, the fluorescent signal from the target pathogen was markedly higher than that of the other pathogens, indicating that successful detection occurs only when the crRNA and target dsDNA are perfectly matched. Therefore, the CRISPR/Cas12a detection system can accurately detect all four diseases of *B. rapa*.

Determining the appropriate concentrations of Cas12a, crRNA, and the ssDNA reporter probe and the optimal cleavage time is crucial for maximizing detection sensitivity. We tested various concentrations of Cas12a protein and crRNA at ratios of 1:0.5, 1:1, 1:1.5, and 1:2. As shown in Fig. S3, the combination of 50 nM crRNA and 50 nM Cas12a achieved the best detection performance. Additionally, we tested different concentrations of five ssDNA reporter probes. As shown in Fig. S4, the ssDNA reporter probe at a concentration of 500 nM yielded the highest detection efficiency. The optimal ratio of Cas12a/crRNA/ssDNA reporter probe concentration was 50 nM:50 nM:500 nM. In these experiments, the reaction time was 1 h. However, the disease levels could be well distinguished within 15 min using the optimal detection system; therefore, 15 min was considered the optimal detection time.

After optimizing the above conditions, we evaluated the sensitivity of the four-disease detection system. A pathogen concentration of 15 ng/μl was used as the starting point for detection, which was serially diluted 10-fold to concentrations of 1.5, 0.15, 0.015, 0.0015, and 0.00015 ng/μl; the results are shown in Fig. 3b. The limit of detection (LOD) was 0.0015 ng/μl for black rot and 0.015 ng/μl for downy mildew, verticillium wilt, and club root. Furthermore, detection sensitivity was influenced by the structural stability of the crRNAs (downy mildew: dG = −6.30; verticillium wilt: dG = −6.80; black rot: dG = −16.30; club root: dG = −9.40). Based on the predicted stability of the crRNAs (Fig. 2) and the results of the detection sensitivity experiment (Fig. 3b), a more stable crRNA structure corresponded to higher detection sensitivity.

In the fluorescence detection platform, we used a LUYOR light as the excitation light source for operation. The FAM-labeled ssDNA probe 1 emits green fluorescence, and the BHQ1-labeled probe 1 shows no fluorescence. When pathogen DNA is present in the system, this DNA will match the crRNA perfectly and form a Cas12a/crRNA/pathogen DNA ternary complex, which activates the trans-cleavage activity of Cas12a, resulting in the cleavage of ssDNA probe 1. Upon irradiation with a blue light source (470–490 nm), it produces green fluorescence: the higher the pathogen content in the system, the more intense the fluorescence. In the absence of pathogen DNA, the crRNA cannot form a ternary complex and cannot activate the trans-cleavage activity of Cas12a, resulting in no fluorescence. Green fluorescence emitted by the FAM-labeled ssDNA probe 1 is absorbed by the BHQ1-labeled probe 1, so no fluorescence is detected when the sample is irradiated with a blue light source. Figure 3c shows the results of visual detection of the four diseases. We irradiated the Cas12a mixture with a blue light source for 5, 10, 15, 30, 45, 60, 75, and 90 min to observe changes in the fluorescence intensity and color of the mixture. Downy mildew pathogen at a concentration of 0.015–15 ng/μl, verticillium wilt and club root pathogens at a concentration of 0.15–15 ng/μl, and black rot pathogen at a concentration of 0.0015–15 ng/μl were distinguishable by the naked eye based on fluorescent signals. The fluorescence intensity stabilized after 15 min, indicating that visual detection of the four diseases can be performed in 15 min.

### Design principle and optimization of the LFB for Cas-AIRPA

**Figure 4:**
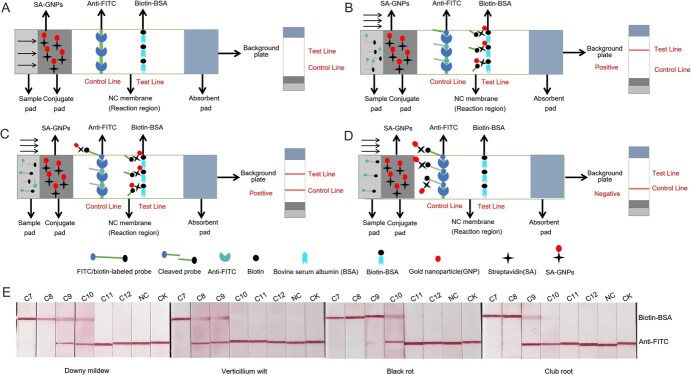
Design principles and sensitivity of the LFB assay for detecting four *B. rapa* diseases. (A) Design of the LFB. (B) and (C) positive results of the LFB assay. (D) Negative results of the LFB assay. (E) Sensitivity of the LFB assay for detecting four diseases. In the sensitivity tests, each pathogen was prepared at concentrations of 15 (C7), 1.5 (C8), 0.15 (C9), 0.015 (C10), 0.0015 (C11), and 0.00015 ng/μl (C12). NC is the negative control, with H_2_O used in place of the DNA template. The DNA template used in the control (CK) was extracted from *B. rapa* leaves without disease. (E) The LFB test results were photographed at the 5-min time point.

The CRISPR/Cas12a-LFB assay was designed based on the trans-cleavage activity of Cas12a and the principle of competition. Figure 4a illustrates the complete LFB, where gold nanoparticles are immobilized with streptavidin (SA-GNPs). The nitrocellulose (NC) membrane contains areas marked as the control line (control line, conjugated with rabbit anti-fluorescein antibody (anti-FITC)) and test line (test line, conjugated with biotinylated bovine serum albumin (biotin-BSA)). Streptavidin-immobilized gold nanoparticles (SA-GNPs) capture biotin label on the probe, regardless of whether the probe has been cleaved, biotin-BSA bind to streptavidin, and anti-FITC capture FITC label on the probe.

When target DNA is present in the sample (Fig. 4a), a ternary complex of Cas12a/crRNA/target DNA forms within the detection system, activating the trans-cleavage activity of Cas12a and nonspecifically cleaves ssDNA probe 3 (Table S2). As the sample flows across the LFB, labeled biotin from the cleaved probe is captured by the SA-GNPs, which are then captured by biotin-BSA on the T line, where SA-GNPs aggregate and appear red; FITC label from the cleaved probe is captured by anti-FITC on the C line. Consequently, the T line appears red while the C line will not (Fig. 4b). If the target DNA content in the sample is low, the probes are not fully cleaved; the biotin label on the intact probe is also captured by SA-GNPs, and the intact probe labeled with FITC is captured by anti-FITC on the C line. As a result, SA-GNPs accumulate at the C line, appearing red. The biotin-labeled cleaved probes are captured by SA-GNPs, which are captured by the biotin-BSA on the T line, causing the SA-GNPs to gather at the T line and appear red. Consequently, both the C and T lines appear red (Fig. 4c).

If no target DNA is present in the sample (Fig. 4d), a ternary complex of Cas12a/crRNA/target DNA does not form within the detection system and the trans-cleavage activity of Cas12a is not activated, allowing the ssDNA probe 3 to remain intact. The intact biotin-labeled probe is captured by SA-GNPs, and the intact FITC-labeled probe is captured by anti-FITC on the C line, where the SA-GNPs accumulate and appear red. Consequently, the C line will appear red and the T line will not.

When designing the LFB, we first optimized the positions of the T line and C line by spraying anti-FITC (1 mg/ml) further from the sample pad than the biotin-BSA (1 mg/ml) (Fig. S5a). In positive samples, only the C line appeared red when the probe was fully cleaved, and both the T line and C line appeared red when the probe was not completely cleaved. In negative samples, both the T line and C line appeared red, resulting in false-positive results. When the positions were reversed, with anti-FITC near the sample pad and biotin-BSA further away (Fig. S5b), positive samples produced a red T line and no C line when the probe was completely cleaved, and T and C lines that were both tinted red when the probe was not completely cleaved. In the negative sample, only the C line appeared red, and the detection results allowing for clear differentiation of the results. Based on these findings, we placed anti-FITC near the sample pad and biotin-BSA farther away as the sample pad as the C line and T line of the LFB, respectively.

We applied 1, 2, 3, 4, and 5 μl SA-GNPs on the sample pad near the NC membrane. As shown in Fig. S6a, the T line appeared red with as little as 3 μl SA-GNPs, and increasing the volume to 4 or 5 μl did not deepen the color. Therefore, we selected 3 μL as the SA-GNP concentration. The interaction between SA-GNPs and the probe influences the chromogenic reaction, meaning that the concentration ratios between the two components affects the result. To optimize the probe concentration in the CRISPR/Cas12a system, we tested five concentrations of ssDNA probe 3 with 3 μl SA-GNPs. As shown in Fig. S6b, probe 3 concentrations between 50 and 250 nM tended to produce false positives, while the chromogenic reaction was weak at 1 μM. Therefore, we selected 500 nM as the optimal probe 3 concentration.

**Figure 5:**
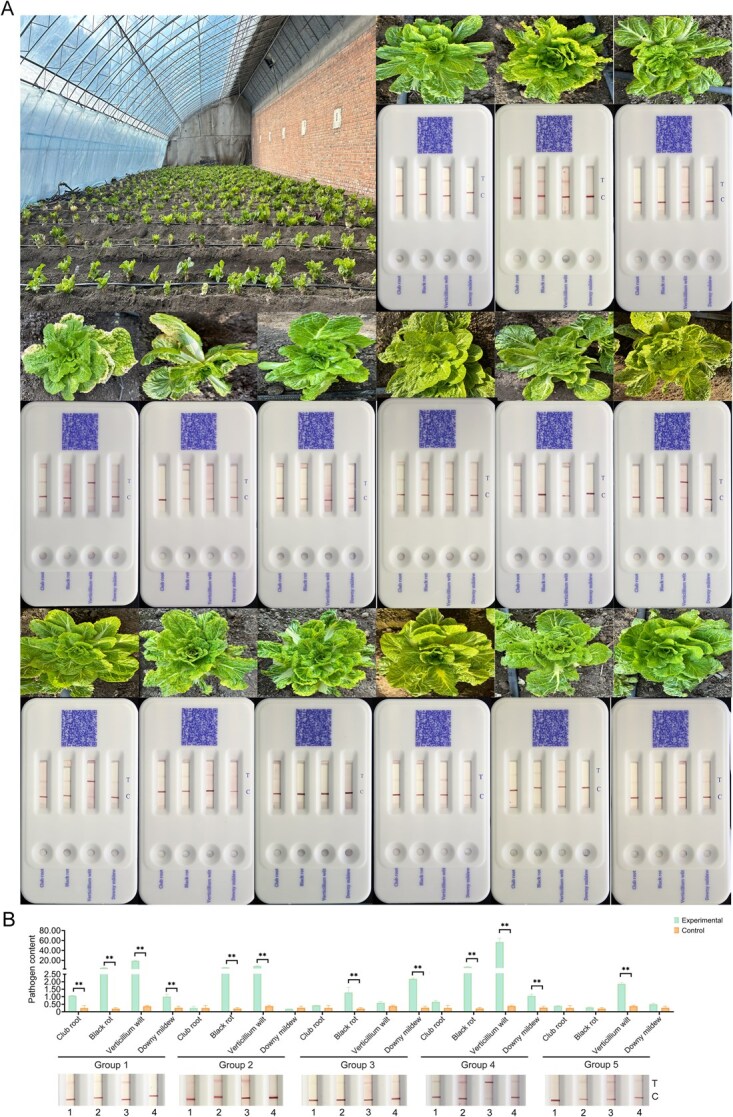
On-spot visual detection of *B. rapa* samples. (A) View of a *B. rapa* field in the BVRC, Beijing, China, and the results of the Cas-AIRPA four-disease test. (B) The results of the first five *B. rapa* samples in (A) examined by RT-qPCR. The order of samples in Fig. 5a is 1–15 from left to right. In Fig. 5b, ‘1’ is club root, ‘2’ is black rot, ‘3’ is verticillium wilt, and ‘4’ is downy mildew. The pathogen content shown on the y-axis refers to the pathogen concentration/*GAPDH* gene concentration.

The chemical properties of the T line and C line significantly influence the adsorption capacity of the NC membrane, thereby affecting its detection ability. To obtain the best detection bands, we optimized the concentrations of anti-FITC and biotin-BSA on the NC membrane. We fixed the concentration of biotin-BSA at 1 mg/ml and tested five concentrations of anti-FITC: 2.5, 2, 1.5, 1, and 0.5 mg/ml. As shown in Fig. S7a, positive samples were not detected using any of these anti-FITC concentrations. We then fixed the concentration of anti-FITC at 1 mg/ml and tested five concentrations of biotin-BSA: 2.5, 2, 1.5, 1, and 0.5 mg/ml. As shown in Fig. S7b, reducing the biotin-BSA concentration enhanced the distinction between positive and negative LFB detection results. Based on these findings, we selected 0.5 mg/ml of biotin-BSA and 1 mg/ml of anti-FITC as the optimal concentrations for the T line and C line, respectively.

After optimizing the above LFB conditions, we evaluated the sensitivity of the four-disease LFBs. A pathogen concentration of 15 ng/μl was used as the initial concentration for the detection of the four diseases and was serially diluted 10-fold to 1.5, 0.15, 0.015, 0.0015, and 0.00015 ng/μl. The results are shown in Fig. 4e. The LOD was 0.0015 ng/μl for black rot, 0.015 ng/μl for downy mildew and clubroot, and 0.15 ng/μl for verticillium wilt.

### Application of Cas-AIRPA in the field

After establishing the Cas-AIRPA platform, we evaluated its applicability for detecting practical samples in the field using a *B. rapa* field in Beijing Vegetable Research Center (BVRC), Beijing, China (Fig. 5). We selected 15 *B. rapa* samples from the field using the five-point sampling method for on-site detection. All samples were analyzed for the presence of downy mildew, verticillium wilt, black rot, and club root; the entire process took ~25 min (Fig. 5a). When detecting verticillium wilt, deep red bands were observed on the T line in 14 samples, indicating that all 14 samples were infected with verticillium wilt and that its incidence was high. When detecting downy mildew, infection was observed in 10 samples; when detecting black rot, infection was observed in 12 samples; when detecting club root, infection was observed in 12 samples. We performed quantitative real-time PCR (qPCR) in the laboratory to verify the detection results of all 15 samples (Fig. 5b and Fig. S8). The results from both detection methods were consistent, demonstrating that the Cas-AIRPA platform is effective for detecting of these four diseases of *B. rapa*.

## Conclusion

**Figure 6:**
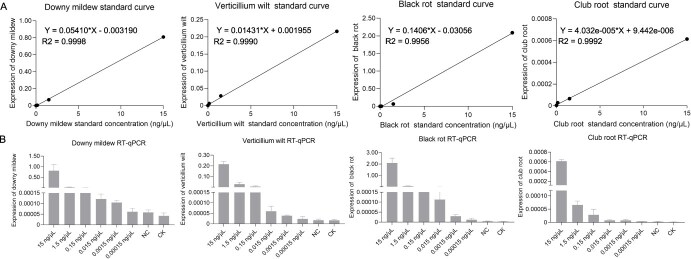
Sensitivity of the RT-qPCR assay for detecting four *B. rapa* diseases. (A) Standard curves of the RT-qPCR assay for detecting four diseases. (B) Sensitivity of the RT-qPCR assay for detecting four diseases. NC is the negative control, with H_2_O used in place of the DNA template. The DNA template in CK was extracted from *B. rapa* leaves without disease. All data were produced from three technical replicates, and data are shown as mean ± SD.

In this study, we developed a visual fluorescence detection platform and a four-channel Cas-AIRPA detection platform for field use. The advantages of fluorescence detection and Cas-AIRPA compared to RT-qPCR detection for *B. rapa* diseases are shown in Table 1. When performing RT-qPCR, we used an initial pathogen concentration of 15 ng/μl for detecting the four diseases, which was serially diluted 10-fold to 1.5, 0.15, 0.015, 0.0015, and 0.00015 ng/μl. An RT-qPCR standard curve was generated (Fig. 6a), and the LOD for RT-qPCR was plotted (Fig. 6b) for each disease. The LOD of downy mildew, verticillium wilt, and black rot was 0.0015 ng/μl, while LOD for club root was 0.15 ng/μl.

**Table 1:** Comparison of fluorescence detection, Cas-AIRPA, and RT-qPCR detection methods.

Methods	RT-qPCR	Visual fluorescence detection	Cas-AIRPA
Specificity	Good	Good	Good
Sensitivity	≥1.5 × 10^−1^ ng/μl	≥1.5 × 10^−1^ ng/μl	≥1.5 × 10^−1^ ng/μl
Accuracy	Good	Good	Good
Test duration	~3 h	~35 min	~25 min
Instrument needed	Yes	No	No
Cost	High	Low	Low

Through the optimization of crRNA structures targeting four pathogens, our detection system achieves high specificity and sensitivity. It can detect downy mildew at concentrations as low as 150 copies/μl, verticillium wilt at 390 copies/μl, black rot at 270 copies/μl, and club root at 550 copies/μl. To facilitate rapid field detection, we utilize the Cas-AIRPA method, enabling the simultaneous identification of these four diseases on-site. The entire process, from DNA extraction to obtaining results, is completed within 25 min without the need for specialized equipment. Furthermore, all diagnostic information can be accessed by scanning the 2D barcode on the quad card with a mobile phone. We believe that with further optimization, this detection system holds great potential for improving multichannel disease detection capabilities in field applications, thereby making a significant contribution on disease prevention.

## Discussion

To achieve disease detection in the field, we integrated rapid DNA extraction and RPA technology with CRISPR/Cas12a detection. The entire process is completed within 25 min without requiring instruments or data analysis. We designed a 4-LFB for the simultaneous detection of the four diseases, which includes a disease-specific 2D barcode that can be scanned with a mobile phone to access relevant information about the diseases detected. Finally, we developed an approach to improving detection sensitivity. We found that optimizing the crRNA structure before synthesis and selecting crRNA with greater stability can enhance detection sensitivity.

CRISPR/Cas12a detection technology has several advantages, including ease of operation, high efficiency, and a short reaction time at room temperature. The integration of CRISPR/Cas12a detection technology with RPA, LAMP, LFBs, and other methods has been successfully used to detect proteins, small molecules, and other substances, with great potential for future applications. However, several challenges hinder the practical use of these techniques, including difficulties in the transporting and storing of Cas12a protein and crRNA, fixing crRNAs for multichannel detection, limited availability of PAM (Protospacer adjacent motif, PAM) sites, and the challenge of achieving quantitative detection with Cas12a. In recent years, the development of high-throughput and multichannel detection methods has become a research focus. CRISPR/Cas12a technology combined with microfluidics can achieve high-throughput, multichannel detection, but the high cost and low portability of microfluidics limit the application of this method. In light of this issue, CRISPR/Cas12a detection technology can be combined with a variety of numerical readout methods, amplification techniques, nanoparticles, internet searches, and artificial intelligence (AI) to continuously develop more efficient, accurate, and stable standardized detection methods.

## Materials and methods

### Materials

The pathogens and diseased plants examined in this study are listed in Table S1. Genomic DNA from the pathogens was provided by the Zhang laboratory, Beijing Academy of Agriculture and Forestry Sciences, China. *Brassica rapa* plants infected with *P. parasitica* (Pers.) Fr., *V. longisporum*, *X. campestris* pv. Campestris (Pammel) Dowson, and *P. brassicae* Woronin were grown in a greenhouse at BVRC.

Healthy plants (*B. rapa* cultivar ‘32’) were grown in a greenhouse in the Zhang laboratory (BVRC). Fresh leaf samples were collected from a farm south of the BVRC, Beijing, China.

Downy mildew genome, 90 Mb; verticillium wilt genome, 35 Mb; black rot genome, 5.1 Mb; club root genome, 25 Mb.

### Nucleic acid extraction

Genomic DNA was extracted from *B. rapa* leaf samples using a rapid DNA extraction method [35]. Fresh leaves (100 mg) were cut into small pieces and transferred into a 1.5-ml centrifuge tube. Using a portable device, 600 μl of extraction buffer (4 M guanidine thiocyanate, 40 mM Tris, 30 mM EDTA, 30 mM Triton X-100, 80 μg/ml RNase A; pH 6.5) was added, and the sample was ground for 1 min. After 30 s of homogenization, the supernatant was filtered through a filtration column connected to a syringe. The filtrate was then passed through an adsorption column containing a silica gel membrane to bind the gDNA. The column was washed sequentially with 350 μl of buffer (4 M guanidine thiocyanate, 40 mM Tris; pH 6.5) and 200 μl of buffer (10 mM Tris, 90 mM NaCl; pH 8.0), discarding the filtrates. Finally, 60 μl of water was added to elute the DNA, which was collected in a new 1.5-ml centrifuge tube. The entire extraction process was completed within 5 min. The DNA was directly subjected to PCR and RPA reactions.

### PCR primers, RPA primers, and probes

PCR primers and RPA primers were designed based on the rDNA-ITS sequences of downy mildew (GenBank: JF975613), verticillium wilt (GenBank: JN564038), black rot (GenBank: CP025750), and club root pathogens (GenBank: AF231027) using Primer3 (https://bioinfo.ut.ee/primer3-0.4.0/). The PCR and RPA primers for downy mildew, verticillium wilt, black rot, and club root are listed in Table S2.

Two fluorescently labeled ssDNA reporter probes (ssDNA probe 1 and ssDNA probe 2; Table S2) were designed for trans-cleavage by CRISPR/Cas12a. ssDNA probe 2 was used to measure the fluorescent signals of the RPA reaction products, and ssDNA probe 1 was used for the visual assessment of the RPA reaction products. ssDNA probe 3 was used for the LFB test in the Cas-AIRPA platform. All primers and reporter probes were purchased from Beijing Tianyi Huiyuan Bioscience & Technology Inc. and Sangon Biotech (Shanghai, China).

### crRNA design and crRNA synthesis

The crRNAs were designed as described previously [36] and are listed in Table S2. The crRNAs were named as follows: for downy mildew, DcrRNA1 and DcrRNA2; for verticillium wilt, WcrRNA1 and WcrRNA2; for black rot, BcrRNA1 and BcrRNA2; and for club root, CcrRNA1 and CcrRNA2. dsDNA templates of the crRNAs were generated by temperature gradient PCR amplification using specific primers for each target gene (D1–1-F/R, D1–2-F/R, D2–1-F/R, and D2–2-F/R for downy mildew; W1–1-F/R, W1–2-F/R, W2–1-F/R, and W2–2-F/R for verticillium wilt; B1–1-F/R, B1–2-F/R, B2–1-F/R, and B2–2-F/R for black rot; C1–1-F/R, C1–2-F/R, C2–1-F/R, and C2–2-F/R for club root). The process involved denaturation at 95°C for 3 min and gradient cooling at 90°C for 30 s; 85°C for 30 s; 80°C for 30 s; 75°C for 30 s; 70°C for 30 s; 65°C for 30 s; 60°C for 30 s; 55°C for 30 s; 50°C for 30 s; 45°C for 30 s; and 40°C for 30 s. After annealing, the reaction was diluted to a concentration of 1 μg/μl. All primers are listed in Table S2. The sgRNAs were synthesized by *in vitro* transcription of the above PCR products using a HiScribe T7 High-Yield RNA Synthesis Kit (New England Biolabs, USA). Finally, the sgRNAs were purified using RNA Clean & Concentrator-25 (Zymo Research) prior to the CRISPR/Cas12a assays.

### Optimization of crRNA structure

The scaffold structure is a ~20-bp sequence upstream of the crRNA, which can fold onto itself and is the key sequence for the binding of Cas protein to the crRNA. Based on the two scaffold structures reported in [35] (scaffold 1, scaffold 2, Table S2), primers were designed corresponding to D1–1 (DcrRNA1 + scaffold 1), D1–2 (DcrRNA1 + scaffold 2), D2–1 (DcrRNA2 + scaffold 1), D2–2 (DcrRNA2 + scaffold 2), W1–1 (WcrRNA1 + scaffold 1), W1–2 (WcrRNA1 + scaffold 2), W2–1 (WcrRNA2 + scaffold 1), W2–2 (WcrRNA2 + scaffold 2), B1–1 (BcrRNA1 + scaffold 1), B1–2 (BcrRNA1 + scaffold 2), B2–1 (BcrRNA2 + scaffold 1), B2–2 (BcrRNA2 + scaffold 2), C1–1 (CcrRNA1 + scaffold 1), C1–2 (CcrRNA1 + scaffold 2), C2–1 (CcrRNA2 + scaffold 1), and C2–2 (CcrRNA2 + scaffold 2). The structural stability of the crRNAs was predicted using the RNA fold web server (http://rna.tbi.univie.ac.at/cgi-bin/RNAWebSuite/RNAfold.cgi), and free energy values (dG) were predicted using RNA Folding Form V2.3 (http://www.unafold.org/mfold/applications/rna-folding-form-v2.php).

### Optimization of the fluorescence detection system

The *in vitro* cleavage reaction of the CRISPR/Cas12a system was performed in a total volume of 20 μl containing 2 μl 10× 2.1 reaction buffer, 1 μl Cas12a (50 nM final), 1 μl crRNA (50 nM final), 1 μl ssDNA probe 1/ssDNA probe 2 (50 nM final), 0.4 μl ROXII (ROXII is not required for visual inspection), 2 μl RPA products, and 12.6 μl ddH_2_O (13 μl ddH_2_O for visual inspection). To detect the reaction products, the cleavage reaction was carried out in a 7500 Real-time PCR system (Thermo Fisher Scientific, USA) at 37°C for 60 min, and the fluorescent signal was monitored every minute. For visual inspection, the fluorescent products were observed under a LUYOR-3415CV fluorescent protein excitation light source (LUYOR, USA).

### Sensitivity and specificity of the fluorescence detection system

To verify the specificity of our detection system, four pathogens were tested (Table S1). To determine the sensitivity, a pathogen concentration of 15 ng/μl was used as the initial concentration for the detection of the four pathogens, followed by serial dilutions by a factor of 10 to 1.5, 0.15, 0.015, 0.0015, and 0.00015 ng/μl (labeled as C1–C6, respectively). Each reaction was performed in triplicate. The calculation formula of pathogen copies are (6.02 × 10^23^ copies/moL) × (pathogen concentrations/pathogen MW).

### Design of the LFB in the Cas-AIRPA platform

The LFB (3 × 60 mm), shown in Fig. 1, includes an absorbent pad, an NC membrane, a conjugate pad, and a sample pad assembled on a plastic adhesive backing card. Anti-FITC (rabbit anti-fluorescein antibody) and biotin-BSA (biotinylated bovine serum albumin) were dispensed onto the NC membrane as the capture reagents. On the NC membrane, areas are marked as the control line (C line) and test line (T line), with the lines separated by 5 mm. SA-GNPs were fixed onto the conjugate pad of the LFB.

The LFB reaction system comprised a total volume of 20 μl containing 2 μl 10× 2.1 reaction buffer, 1 μl Cas12a (50 nM final), 1 μl crRNA (50 nM final), 1 μl ssDNA probe 3 (50 nM final), 2 μl RPA products, and 13 μl ddH_2_O. A 5-μl aliquot of the CRISPR-Cas12a reaction mixture was added to the sample region of the LFB, followed by the addition of 50 μl PBS buffer (0.1 M PBS, pH 7.4); the results were visualized as red bands on the NC membrane within 2 min.

### Sensitivity of the Cas-AIRPA assays

To determine the sensitivity of the Cas-AIRPA assays, a pathogen concentration of 15 ng/μl was used as the initial concentration for the detection of the four pathogens, followed by serial dilutions by a factor of 10 to 1.5, 0.15, 0.015, 0.0015, and 0.00015 ng/μl (labeled C7–C12, respectively).

### Design of a device for simultaneous disease detection using the Cas-AIRPA platform

A quad card was designed with four detection channels to detect downy mildew, verticillium wilt, black rot, and club root, and a disease-specific 2D barcode at the top that can be scanned with a mobile phone (Fig. 1). The first page of the file accessed by scanning the 2D barcode contains diagnostic information about the disease and a link to detailed information about each disease (in the form of a button). This information includes descriptions of disease symptoms and pathogens, photographs of disease symptoms, crRNA sequences for the detection sites, and rDNA-ITS sequences of the pathogens. Jiaxing Lilan Biotechnology Co., Ltd., produced the quad card and 2D barcodes according to our design. The usage and contents accessed via the 2D barcode are shown in the Supplementary Video.

### Applicability of the Cas-AIRPA platform for the detection of *B. rapa* diseases in the field

To evaluate the utility of the Cas-AIRPA platform, a five-point sampling method was used to select 15 *B. rapa* leaf samples from greenhouse-grown plants for disease detection. The Cas-AIRPA platform was used for the entire disease detection process, including rapid DNA extraction, RPA, the LFB, and the quad card. In addition, RT-qPCR was performed on all samples to validate the results of the Cas-AIRPA analysis.

## Supplementary Material

Web_Material_uhae351

## Data Availability

Data and Materials Availability. All data needed to evaluate the conclusions in the paper are present in the paper and/or the Supplementary Materials. All materials generated in this study are available from the corresponding author S.C.Y.

## References

[1] Dong H, Xiong R, Liang Y. et al. Development of glycine-copper(II) hydroxide nanoparticles with improved biosafety for sustainable plant disease management. RSC Adv. 2020;10:21222–7 35518721 10.1039/d0ra02050h PMC9054364

[2] Su T, Wang W, Wang Z. et al. BrMYB108 confers resistance to verticillium wilt by activating ROS generation in Brassica rapa. Cell Rep. 2023;42:112938 37552600 10.1016/j.celrep.2023.112938

[3] Sundelin T, Jensen DF, Lübeck M. Identification of expressed genes during infection of Chinese cabbage (Brassica rapa subsp. pekinensis) by Plasmodiophora brassicae. J Eukaryot Microbiol. 2011;58:310–4 21518080 10.1111/j.1550-7408.2011.00551.x

[4] Zhang B, Su T, Xin X. et al. Wall-associated kinase BrWAK1 confers resistance to downy mildew in Brassica rapa. Plant Biotechnol J. 2023;21:2125–39 37402218 10.1111/pbi.14118 PMC10502744

[5] Zhang B, Li P, Su T. et al. BrRLP48, encoding a receptor-like protein, involved in downy mildew resistance in Brassica rapa. Front Plant Sci. 2018;9:1708 30532761 10.3389/fpls.2018.01708 PMC6265505

[6] Singh D, Dhar S, Yadava DK. Genetic and pathogenic variability of Indian strains of Xanthomonas campestris pv. campestris causing black rot disease in crucifers. Curr Microbiol. 2011;63:551–60 21956666 10.1007/s00284-011-0024-0

[7] Ahmed A, Munir S, He P. et al. Biocontrol arsenals of bacterial endophyte: an imminent triumph against clubroot disease. Microbiol Res. 2020;241:126565 32829185 10.1016/j.micres.2020.126565

[8] Khater M, de la Escosura-Muñiz A, Merkoçi A. Biosensors for plant pathogen detection. Biosens Bioelectron. 2017;93:72–86 27818053 10.1016/j.bios.2016.09.091

[9] Querci M, Van den Bulcke M, Žel J. et al. New approaches in GMO detection. Anal Bioanal Chem. 2010;396:1991–2002 19876618 10.1007/s00216-009-3237-3

[10] Datukishvili N, Kutateladze T, Gabriadze I. et al. Newmultiplex PCR methods for rapid screening of genetically modified organisms in foods. Front Microbiol. 2015;6:757 26257724 10.3389/fmicb.2015.00757 PMC4513241

[11] Patwardhan S, Dasari S, Bhagavatula K. et al. Simultaneous detection of genetically modified organisms in a mixture by multiplex PCR-Chip capillary electrophoresis. J AOAC Int. 2015;98:1366–74 26525256 10.5740/jaoacint.15-070

[12] Dobnik D, Spilsberg B, Bogožalec Košir A. et al. Multiplex quantification of 12 European Union authorized genetically modified maize lines with droplet digital polymerase chain reaction. Anal Chem. 2015;87:8218–26 26169291 10.1021/acs.analchem.5b01208

[13] Berg T, Tesoriero L, Hailstones DL. A multiplex real-time PCR assay for detection of Xanthomonas campestris from brassicas. Lett Appl Microbiol. 2006;42:624–30 16706903 10.1111/j.1472-765X.2006.01887.x

[14] Treml D, Venturelli GL, Brod FCA. et al. Development of an event-specific hydrolysis probe quantitative real-time polymerase chain reaction assay for Embrapa 5.1 genetically modified common bean (Phaseolus vulgaris). J Agric Food Chem. 2014;62:11994–2000 25437743 10.1021/jf503928m

[15] Venturelli GL, Brod FCA, Rossi GB. et al. A specific endogenous reference for genetically modified common bean (Phaseolus vulgaris L.) DNA quantification by real-time PCR targeting lectin gene. Mol Biotechnol. 2014;56:1060–8 25078400 10.1007/s12033-014-9786-5

[16] Chen X, Wang X, Jin N. et al. Endpoint visual detection of three genetically modified rice events by loop-mediated isothermal amplification. Int J Mol Sci. 2012;13:14421–33 23203072 10.3390/ijms131114421 PMC3509588

[17] Huang X, Zhai C, You Q. et al. Potential of cross-priming amplification and DNA-based lateral-flow strip biosensor for rapid on-site GMO screening. Anal Bioanal Chem. 2014;406:4243–9 24736809 10.1007/s00216-014-7791-y

[18] Wu H, Zhang X, Wu B. et al. Rapid on-site detection of genetically modified soybean products by realtime loop-mediated isothermal amplification coupled with a designed portable amplifier. Food Chem. 2020;323:126819 32334306 10.1016/j.foodchem.2020.126819

[19] Zhu Z, Li R, Zhang H. et al. PAM-free loop-mediated isothermal amplification coupled with CRISPR/Cas12a cleavage (Cas-PfLAMP) for rapid detection of rice pathogens. Biosens Bioelectron. 2022;204:114076 35180691 10.1016/j.bios.2022.114076

[20] Liu H, Wang J, Zeng H. et al. RPA-Cas12a-FS: a frontline nucleic acid rapid detection system for food safety based on CRISPR-Cas12a combined with recombinase polymerase amplification. Food Chem. 2021;334:127608 32711280 10.1016/j.foodchem.2020.127608

[21] Zeng H, Wang J, Jia J. et al. Development of a lateral flow test strip for simultaneous detection of BTCry1Ab, BT-Cry1Ac and CP4 EPSPS proteins in genetically modified crops. Food Chem. 2021;335:127627 32738534 10.1016/j.foodchem.2020.127627

[22] Son C-Y, Haines BB, Luch A. et al. Identification of the transgenic integration site in 2C T cell receptor transgenic mice. Transgenic Res. 2018;27:441–50 30132177 10.1007/s11248-018-0090-1

[23] Tengs T, Kristoffersen AB, Berdal KG. et al. Microarray-based method for detection of unknown genetic modifications. BMC Biotechnol. 2007;7:91 18088429 10.1186/1472-6750-7-91 PMC2225397

[24] Evangelista VO, Pelegrini PB, Mulinari F. et al. A novel immunocromatographic strip test for rapid detection of Cry1Ac and Cry8Ka5 proteins in genetically modified crops. Anal Methods. 2015;7:9331–9

[25] Li Y, Li S, Wang J. et al. CRISPR/Cas systems towards next-generation biosensing. Trends Biotechnol. 2019;37:730–43 30654914 10.1016/j.tibtech.2018.12.005

[26] Sashital DG . Pathogen detection in the CRISPR–Cas era. Genome Med. 2018;10:32 29690921 10.1186/s13073-018-0543-4 PMC5937823

[27] Fonfara I, Richter H, Bratovič M. et al. The CRISPR-associated DNA-cleaving enzyme Cpf1 also processes precursor CRISPR RNA. Nature. 2016;532:517–21 27096362 10.1038/nature17945

[28] He Q, Yu D, Bao M. et al. High-throughput and all-solution phase African swine fever virus (ASFV) detection using CRISPR-Cas12a and fluorescence based point-of-care system. Biosens Bioelectron. 2020;154:112068 32056963 10.1016/j.bios.2020.112068

[29] Huifeng X, Rui P, Weihua H. et al. Label-free dual-mode sensing platform based on target-regulated CRISPR–Cas12a activity for ochratoxin A in Morinda officinalis. Anal Methods. 2023;15:4518–23 37622284 10.1039/d3ay01025b

[30] Gootenberg JS, Abudayyeh OO, Lee JW. et al. Nucleic acid detection with CRISPR-Cas13a/C2c2. Science. 2017;356:438–42 28408723 10.1126/science.aam9321 PMC5526198

[31] Shen J, Zhou X, Shan Y. et al. Sensitive detection of a bacterial pathogen using allosteric probe-initiated catalysis and CRISPRCas13a amplification reaction. Nat Commun. 2020;11:267 31937772 10.1038/s41467-019-14135-9 PMC6959245

[32] Smargon AA, Cox DBT, Pyzocha NK. et al. Cas13b is a type VI-B CRISPR-associated RNA-guided RNAse differentially regulated by accessory proteins Csx27 and Csx28. Mol Cell. 2017;65:618–630.e7 28065598 10.1016/j.molcel.2016.12.023 PMC5432119

[33] Li S-Y, Cheng Q-X, Wang J-M. et al. CRISPR-Cas12a-assisted nucleic acid detection. Cell Discov. 2018;4:20 29707234 10.1038/s41421-018-0028-z PMC5913299

[34] Chen JS, Ma E, Harrington LB. et al. CRISPR-Cas12a target binding unleashes indiscriminate single-stranded DNase activity. Science. 2018;360:436–9 29449511 10.1126/science.aar6245 PMC6628903

[35] Wang M, Liu X, Yang J. et al. CRISPR/Cas12a-based biosensing platform for the on-site detection of single-base mutants in gene-edited rice. Front Plant Sci. 2022;13:944295 36161021 10.3389/fpls.2022.944295 PMC9490305

[36] Zetsche B, Gootenberg JS, Abudayyeh OO. et al. Cpf1 is a single RNA-guided endonuclease of a class 2 CRISPRCas system. Cell. 2015;163:759–71 26422227 10.1016/j.cell.2015.09.038 PMC4638220

