# Supplementary material for: A multi-channel CRISPR-based method for rapid, sensitive detection of four diseases of Brassica rapa in the field
Source: Hortic Res. 2024 Dec 12;12(3):uhae351. doi: 10.1093/hr/uhae351 (PMC11890027; doi:10.1093/hr/uhae351)
Supplement: Web_Material_uhae351 [file web_material_uhae351.zip › Supplementary Materials.docx]

**Supplementary Figures**


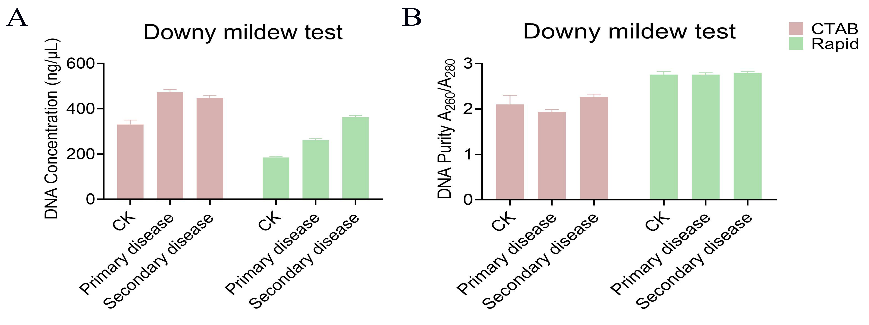


Figure S1 Comparison of the concentration and purity of DNA extracted by rapid nucleic acid extraction and the CTAB method

1. . Comparison of the concentration of DNA extracted method by rapid nucleic acid extraction and the CTAB method. (B). Comparison of the purity of DNA extracted by rapid nucleic acid extraction and the CTAB method. Leaves of plants with primary disease (downy mildew) symptoms, secondary disease (downy mildew) symptoms, and leaves of plants without disease symptoms (CK) were selected for DNA extraction using the two methods.


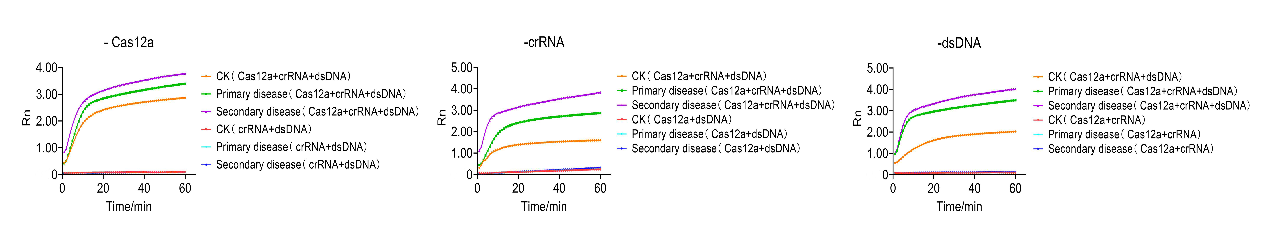


Figure S2 Validation of the detection activity of the CRISPR/Cas12a system

In the fluorescence detection experiment, leaves with different levels of disease severity (primary disease and secondary disease, downy mildew) and leaves without disease (CK) were selected for the CRISPR/Cas activity test. The fluorescent signal was monitored and recorded every minute for a total of 60 minutes. All data were produced from three technical replicates, and data are shown as mean±SD.


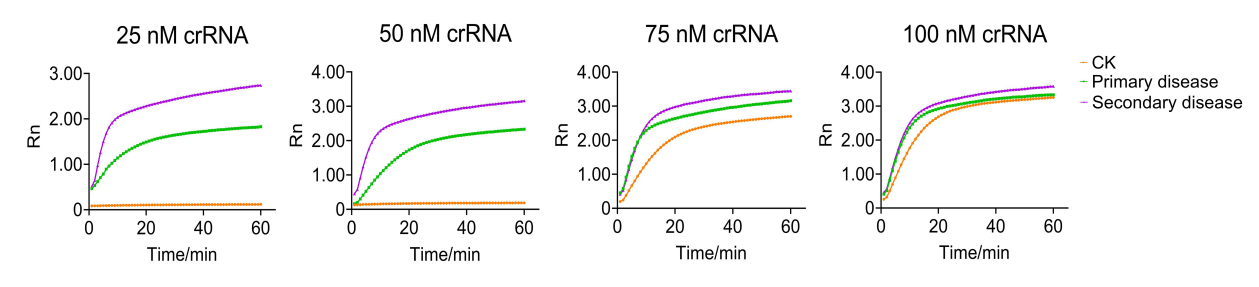


Figure S3 Optimization of crRNA concentration in the CRISPR/Cas12a detection system

Leaves with different levels of disease severity (primary disease and secondary disease, downy mildew) and leaves without disease symptoms (CK) were selected to test the optimal crRNA concentration in the CRISPR/Cas system. The fluorescent signal was monitored and recorded every minute for a total of 60 minutes. All data were produced from three technical replicates, and data are shown as mean±SD.


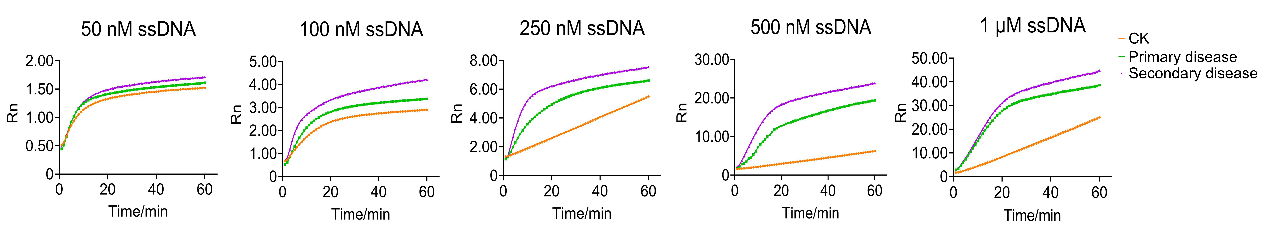


Figure S4 Optimization of ssDNA reporter probe concentration in the CRISPR/Cas12a detection system

Leaves with different levels of disease severity (primary disease and secondary disease, downy mildew) and leaves without disease symptoms (CK) were selected to optimize the ssDNA reporter probe concentration in the CRISPR/Cas system. The fluorescent signal was monitored and recorded every minute for a total of 60 minutes. All data were produced from three technical replicates, and data are shown as mean±SD.

**
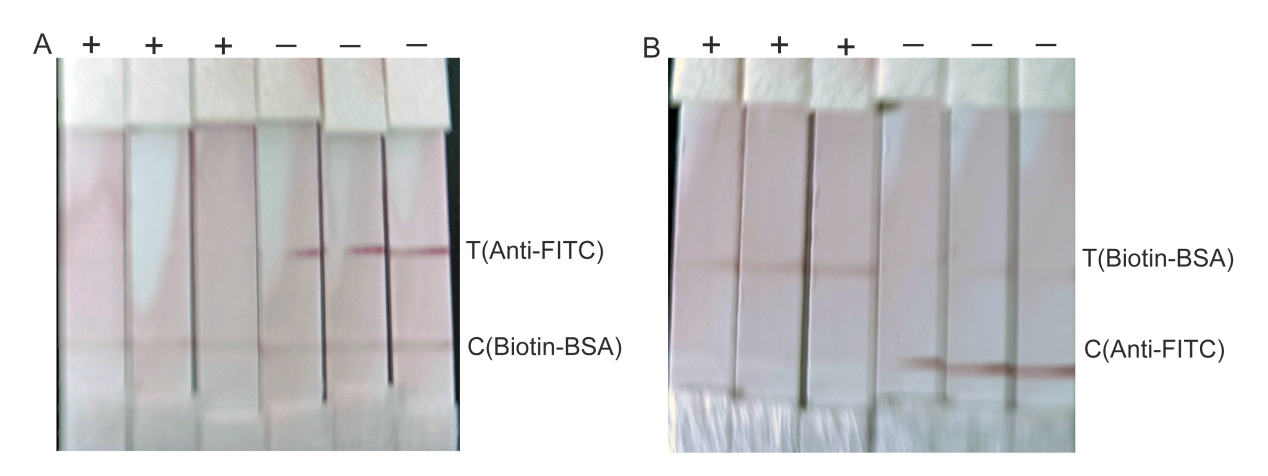
**

Figure S5 Selection of the locations of the C and T lines in the LFB

(A). Biotin-BSA was sprayed near the sample pad and anti-FITC was sprayed away from the sample pad; (B). Anti-FITC was sprayed near the sample pad and biotin-BSA was sprayed away from the sample pad. +, Positive sample; –, Negative sample.


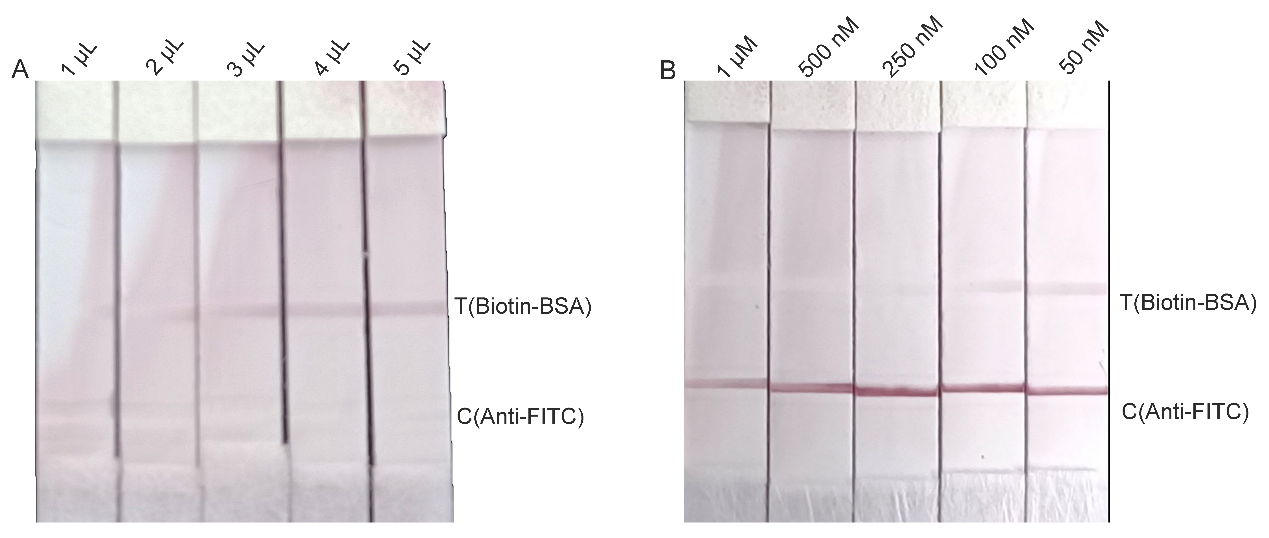


Figure S6 Optimization of SA-GNPs and ssDNA probe 3 concentrations in the LFB

(A). Optimization of SA-GNPs concentration; (B). Optimization of ssDNA probe 3 concentration.


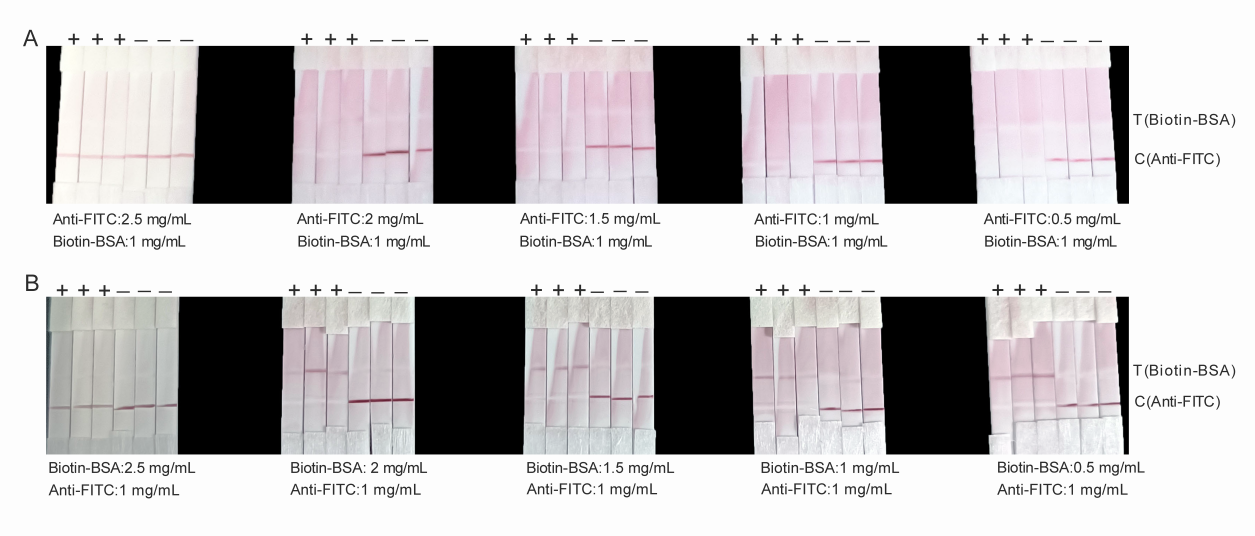


Figure S7 Optimization of biotin-BSA and anti-FITC concentrations in the C and T lines in the LFB

(A). The fixed biotin-BSA concentration was 1 mg/mL, and the concentrations of anti-FITC (from left to right) were 2.5, 2, 1.5, 1, and 0.5 mg/mL. (B). The fixed anti-FITC concentration was 1 mg/mL, and the concentrations of biotin-BSA (from left to right) were 2.5, 2, 1.5, 1, and 0.5 mg/mL. +, Positive sample; –, Negative sample.


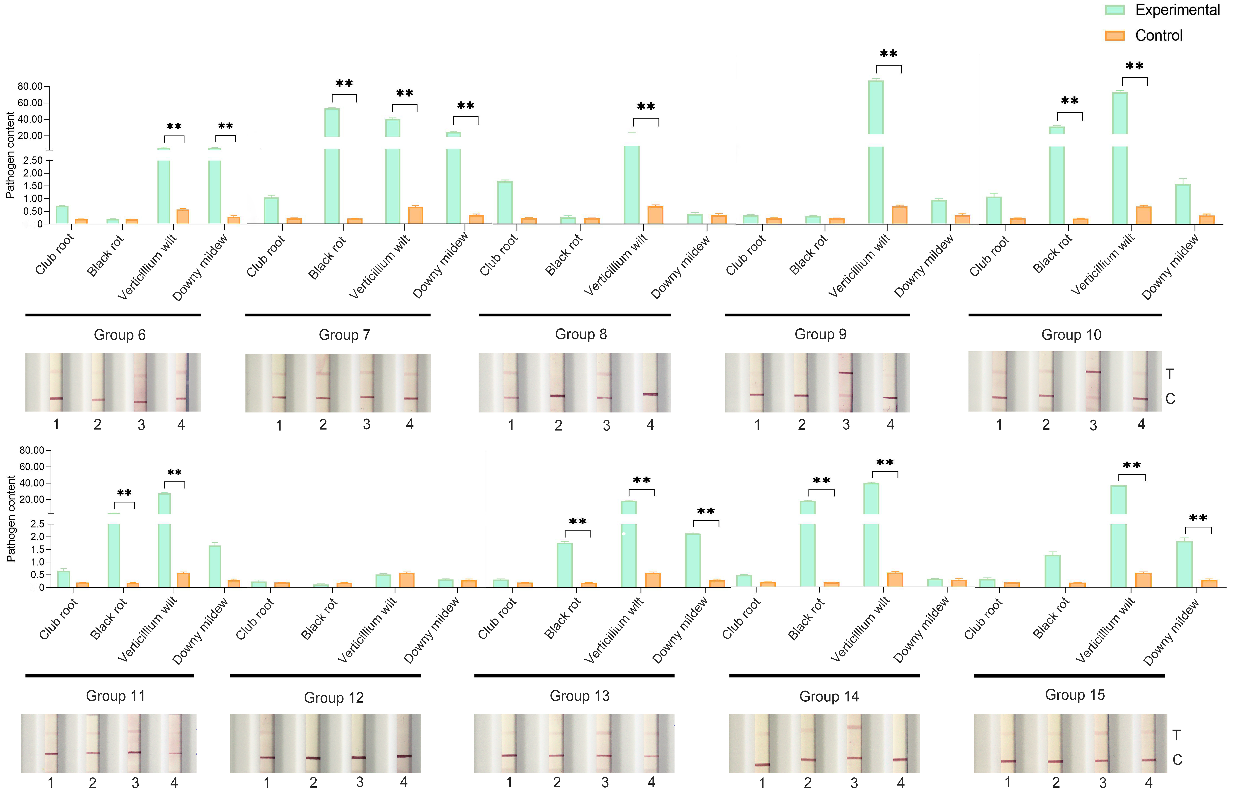


Figure S8 Four diseases of *B. rapa* samples were detected by RT-qPCR

The results of the remaining ten *B. rapa* samples in Figure 5A examined by RT-qPCR. The order of samples in Figure 5A is 1 to 15 from left to right. In Figure S8, “1” is club root, “2” is black rot, “3” is verticillium wilt, and “4” is downy mildew. The pathogen content shown on the y-axis refers to the pathogen concentration/*GAPDH* gene concentration.

**Supplementary video**

**Supplementary tables**

Table S1 Information about the pathogens analyzed in this study

| Pathogen | Latin name | Sequence source |
| --- | --- | --- |
| Downy mildew | *Peronospora parasitica* (Pers.) Fr | GenBank: JF975613 |
| Verticillium wilt | *Verticillium dahliae* Klebahu | GenBank: JN564038 |
| Black rot | *Xanthomonas campestris* pv. campestris (Pammel) Dowson | GenBank: CP025750 |
| Club root | *Plasmodiophora brassicae* Woronin | GenBank: AF231027 |

Table S2 Primers used in this study

| Name | Primer sequence (5’-3’) |
| --- | --- |
| RPA-Downy-ITS-F | TCCGTAGGTGAACCTGCGGAAGGATCATTACCA |
| RPA-Downy-ITS-R | CGCATTACGTATCGCAATTCGCAGCGTTCTTCATC |
| RPA-Wilt-ITS-F | GTAGGTGAACCTGCGGAGGGATCATTACCGAGT |
| RPA-Wilt-ITS-R | TCGAGGGTTGAAACGACGCTCGGACAGGCAT |
| RPA-Black-ITS-F | TGGACCGTCGCCCCGATCGTCGCCTGTTCAGC |
| RPA-Black-ITS-R | ACCGCGAATCGCCACGCATGCTTTCCCACTGT |
| RPA-Club-ITS-F | TTGGGCTCTTAGAAGAAGGAGAAGTCGTAAC |
| RPA-Club-ITS-R | CCTTGTCCGTTGTTACCATACCCAGGGCGATT |
| DNA-Downy-ITS-F | GAACCTGCGGAAGGATCA |
| DNA-Downy-ITS-R | AATTCGCAGCGTTCTTCATC |
| DNA-Wilt-ITS-F | CGGAGGGATCATTACCGAGT |
| DNA-Wilt-ITS-R | AAACGACGCTCGGACAGG |
| DNA-Black-ITS-F | GATCGTCGCCTGTTCAGC |
| DNA-Black-ITS-R | ACGCATGCTTTCCCACTGT |
| DNA-Club-ITS-F | CGGAGTGGTCGAACTTCATT |
| DNA-Club-ITS-R | GTTACCATACCCAGGGCGAT |
| DcrRNA1 | TTTCCACGTGAACCGTATTAACAA |
| DcrRNA2 | TTTGGTGAAGTAGTGAAAATTTTA |
| WcrRNA1 | TTTGTGAACCATATTGTTGCTTCG |
| WcrRNA2 | TTTATACCAACGATACTTCTGAGT |
| BcrRNA1 | TTTCCACTGTTACAGCGTGGCCGA |
| BcrRNA2 | TTTCCAGCGCCGTCGCCGGCGCGG |
| CcrRNA1 | TTTCCGTAGGCGTCGTCGTCGGCG |
| CcrRNA2 | TTTGTCTTACCAAGTGCTTGTGCG |
| Scaffold 1 | UAAUUUCUACUAAGUGUAGAU |
| Scaffold 2 | AAUUUCUACUGUUGUAGAU |
| D1-1-F | GAAATTAATACGACTCACTATAGGGTAATTTCTACTAAGTGTAGATCACGTGAACCGTATTAACAA |
| D1-1-R | TTGTTAATACGGTTCACGTGATCTACACTTAGTAGAAATTACCCTATAGTGAGTCGTATTAATTTC |
| D1-2-F | GAAATTAATACGACTCACTATAGGGAATTTCTACTGTTGTAGATCACGTGAACCGTATTAACAA |
| D1-2-R | TTGTTAATACGGTTCACGTGATCTACAACAGTAGAAATTCCCTATAGTGAGTCGTATTAATTTC |
| D2-1-F | GAAATTAATACGACTCACTATAGGGTAATTTCTACTAAGTGTAGATGTGAAGTAGTGAAAATTTTA |
| D2-1-R | TAAAATTTTCACTACTTCACATCTACACTTAGTAGAAATTACCCTATAGTGAGTCGTATTAATTTC |
| D2-2-F | GAAATTAATACGACTCACTATAGGGAATTTCTACTGTTGTAGATGTGAAGTAGTGAAAATTTTA |
| D2-2-R | TAAAATTTTCACTACTTCACATCTACAACAGTAGAAATTCCCTATAGTGAGTCGTATTAATTTC |
| W1-1-F | GAAATTAATACGACTCACTATAGGGTAATTTCTACTAAGTGTAGATTGAACCATATTGTTGCTTCG |
| W1-1-R | CGAAGCAACAATATGGTTCAATCTACACTTAGTAGAAATTACCCTATAGTGAGTCGTATTAATTTC |
| W1-2-F | GAAATTAATACGACTCACTATAGGGAATTTCTACTGTTGTAGATTGAACCATATTGTTGCTTCG |
| W1-2-R | CGAAGCAACAATATGGTTCAATCTACAACAGTAGAAATTCCCTATAGTGAGTCGTATTAATTTC |
| W2-1-F | GAAATTAATACGACTCACTATAGGGTAATTTCTACTAAGTGTAGATTACCAACGATACTTCTGAGT |
| W2-1-R | ACTCAGAAGTATCGTTGGTAATCTACACTTAGTAGAAATTACCCTATAGTGAGTCGTATTAATTTC |
| W2-2-F | GAAATTAATACGACTCACTATAGGGAATTTCTACTGTTGTAGATTACCAACGATACTTCTGAGT |
| W2-2-R | ACTCAGAAGTATCGTTGGTAATCTACAACAGTAGAAATTCCCTATAGTGAGTCGTATTAATTTC |
| B1-1-F | GAAATTAATACGACTCACTATAGGGTAATTTCTACTAAGTGTAGATCACTGTTACAGCGTGGCCGA |
| B1-1-R | TCGGCCACGCTGTAACAGTGATCTACACTTAGTAGAAATTACCCTATAGTGAGTCGTATTAATTTC |
| B1-2-F | GAAATTAATACGACTCACTATAGGGAATTTCTACTGTTGTAGATCACTGTTACAGCGTGGCCGA |
| B1-2-R | TCGGCCACGCTGTAACAGTGATCTACAACAGTAGAAATTCCCTATAGTGAGTCGTATTAATTTC |
| B2-1-F | GAAATTAATACGACTCACTATAGGGTAATTTCTACTAAGTGTAGATCAGCGCCGTCGCCGGCGCGG |
| B2-1-R | CCGCGCCGGCGACGGCGCTGATCTACACTTAGTAGAAATTACCCTATAGTGAGTCGTATTAATTTC |
| B2-2-F | GAAATTAATACGACTCACTATAGGGAATTTCTACTGTTGTAGATCAGCGCCGTCGCCGGCGCGG |
| B2-2-R | CCGCGCCGGCGACGGCGCTGATCTACAACAGTAGAAATTCCCTATAGTGAGTCGTATTAATTTC |
| C1-1-F | GAAATTAATACGACTCACTATAGGGTAATTTCTACTAAGTGTAGATCGTAGGCGTCGTCGTCGGCG |
| C1-1-R | CGCCGACGACGACGCCTACGATCTACACTTAGTAGAAATTACCCTATAGTGAGTCGTATTAATTTC |
| C1-2-F | GAAATTAATACGACTCACTATAGGGAATTTCTACTGTTGTAGATCGTAGGCGTCGTCGTCGGCG |
| C1-2-R | CGCCGACGACGACGCCTACGATCTACAACAGTAGAAATTCCCTATAGTGAGTCGTATTAATTTC |
| C2-1-F | GAAATTAATACGACTCACTATAGGGTAATTTCTACTAAGTGTAGATTCTTACCAAGTGCTTGTGCG |
| C2-1-R | CGCACAAGCACTTGGTAAGAATCTACACTTAGTAGAAATTACCCTATAGTGAGTCGTATTAATTTC |
| C2-2-F | GAAATTAATACGACTCACTATAGGGAATTTCTACTGTTGTAGATTCTTACCAAGTGCTTGTGCG |
| C2-2-R | CGCACAAGCACTTGGTAAGAATCTACAACAGTAGAAATTCCCTATAGTGAGTCGTATTAATTTC |
| ssDNA probe 1 | 6-FAM-TTTTTTTT- BHQ1 |
| ssDNA probe 2 | 6-FAM-TTTTTTTT- TAMRA |
| ssDNA probe 3 | 6-FITC-TTTTTTTT-Biotin |
